# Behavioral and electrophysiological findings of central auditory processing in children and adolescents undergoing musical training

**DOI:** 10.1590/2317-1782/e20240318en

**Published:** 2026-04-27

**Authors:** Bruna de Franceschi Schirmer Gindri, Tainara Milbradt Weich Wagner, Ana Cláudia Figueiredo Frizzo, Eliara Pinto Vieira Biaggio

**Affiliations:** 1 Departamento de Fonoaudiologia, Universidade Federal de Santa Maria – UFSM - Santa Maria (RS), Brasil.; 2 Programa de Pós-graduação em Fonoaudiologia, Universidade Estadual Paulista Julio de Mesquita Filho – UNESP - São Paulo (SP), Brasil.

**Keywords:** Child, Adolescent, Hearing, Auditory Perceptual Disorders, Neuronal Plasticity

## Abstract

**Purpose:**

This study analyzes the outcomes of behavioral and electrophysiological assessments of central auditory processing in children and adolescents undergoing musical training through clinical protocols or structured educational methods.

**Research strategies:**

A systematic search was conducted in three electronic databases (PubMed, Web of Science, and Scopus) between April and June 2024. Two researchers independently applied the following search terms combined with the Boolean operator "AND": "musical training," "child," and "adolescent."

**Selection criteria:**

The PICOS framework guided the selection process, focusing on population (children and adolescents), intervention (musical training), comparison (with non-trained groups), outcome (central auditory processing effects), and study design (cohort, case-control, and randomized clinical trials).

**Data analysis:**

Data were extracted and tabulated by the researchers and analyzed descriptively. Methodological quality was assessed using the NHLBI Quality Assessment Tool.

**Results:**

Among 800 initial publications, five duplicates were removed. Of the remaining 795 studies, 783 did not meet the inclusion criteria. A full-text review of 12 studies led to the exclusion of one due to unavailability and four due to quality issues. Ultimately, seven articles were included.

**Conclusion:**

The results highlight improvements in sound discrimination, speech-in-noise processing, environmental sound change detection, and selective attention. These findings underscore the necessity of avoiding overly brief training programs.

## INTRODUCTION

Hearing is a primary sense for human beings, crucial for language acquisition and the proper development of speech during childhood. It is also a vital means for understanding the world^(1,2)^. The efficiency and effectiveness with which the central auditory nervous system (CANS) processes auditory information are referred to as central auditory processing (CAP)^(3)^. If this process does not occur adequately, it can lead to central auditory processing disorder, resulting in communication deficits and other functional impairments that depend on the age at which the disorder manifests^(4)^.

It is important to highlight the close relationship between hearing and language in childhood, as the appropriate development of the perception of complex sounds, language acquisition, and proper speech production relies on the effective integration of auditory information by the central nervous system. Moreover, speech production involves cortical and subcortical sensory and motor networks, integrating auditory information and sound representation, ultimately leading to the motor actions responsible for the emission of speech sounds^(5)^.

Childhood is a critical period for identifying changes in central auditory abilities, coinciding with the peak maturation of the CANS and when central auditory processing disorders can have the greatest impact^(6)^. Studies have highlighted the positive impact of musical training on auditory perception^(7-11)^. Musical practice and training enhance specific CAP skills at various developmental stages^(12)^. Exposure to music, even in newborns, influences auditory information learning and brain development^(13,14)^. Furthermore, musical training in children has been demonstrated to accelerate the maturation of central auditory skills^(15-17)^.

Simply put, there is growing evidence supporting the use of music to stimulate brain areas related to auditory and language processing^(18)^, as well as to improve physical and emotional well-being^(19)^. Hence, music positively influences the overall development of children, enhancing not only communicative skills, but also metalinguistic and auditory abilities^(5)^. Understanding these effects may contribute to developing more effective therapeutic strategies and educational policies aimed at supporting children with central auditory processing disorder or at risk for such disorders. Additionally, investigating the impact of different types of musical training and considering their variables may help clarify the mechanisms through which music influences auditory and language development.

Given the above, this systematic review aimed to examine behavioral and electrophysiological studies of CAP in children and adolescents who have participated in musical training through clinical protocols or structured educational programs in both public and private school settings.

## METHODS

This systematic review examined the impact of musical training on CAP in children and adolescents. Musical training was defined broadly to include both clinical/protocol approaches and structured educational programs in public or private academic settings, reflecting its widespread curricular integration in many developed countries. The review complied with the Preferred Reporting Items for Systematic Reviews and Meta-Analyses (PRISMA) checklist^(20)^, and the protocol of this review was duly registered in the Open Science Framework (OSF)^(21)^.

### Inclusion criteria

The review focused on the behavioral and electrophysiological findings of CAP in the target population, applying PICOS criteria: population (children and adolescents aged 5–18 years), intervention (musical training through clinical protocols or structured educational programs in public and private schools) comparison (between those undergoing musical training and those who are not), outcome (effects on CAP), and Study design (cohort, case-control, and randomized clinical trials). Exclusions were systematic and narrative reviews, observational case reports/series, abstracts, studies off-topic, lacking explicitness, or failing to address the research question.

The studies evaluated were those without language or period restrictions, which contained information on the behavioral and electrophysiological findings of CAP in children and adolescents undergoing musical training. To be included in this review, studies were required to score above 80% on the Study Quality Assessment Tools of the National Heart, Lung, and Blood Institute (NHLBI)^(22)^ to ensure high quality.

### Search strategy and study selection

Researchers conducted searches in PubMed, Web of Science, and Scopus from April to June 2024, using a consistent strategy and the terms "musical training" AND "child" AND "adolescent" with no restrictions on age, publication year, or language.

It is important to highlight that in this research strategy, terms directly related to central auditory processing were not used, which could potentially limit the findings by preventing important articles from being included in this review. Therefore, terms directly linked to the intervention and the target audience of this review were utilized.

The articles were independently and blindly selected by two researchers, minimizing the risk of bias. The preliminary screening involved reviewing titles and abstracts based on inclusion criteria. The authors' and journals’ names were concealed to prevent bias and conflicts of interest. The final selection involved a thorough review of the full articles.

Articles deemed irrelevant or whose full-text was unavailable were excluded. Throughout this process, any discrepancies were resolved through peer discussions; when a consensus could not be reached, a third researcher was consulted for a decisive verdict.

### Quality analysis and analysis of the results

Each study was evaluated independently by the authors. The overall quality was ranked as follows: scores above 80% indicated good quality, 50–79% were considered fair, and <50% were deemed poor. Only studies with scores above 80% were included in this review. To evaluate the quality of articles, the NHLBI Quality Assessment Tool^(22)^ was employed, which includes 14 items to be assessed according to the type of study analyzed (Chart 1). Regarding the responses to the inquiries, the options were ‘Yes,’ ‘No,’ ‘Cannot determine’ (CD), Not applicable (NA), and Not reported (NR). In the translation of the table, 'not informed' is uniformly used, indicating that to achieve 80% quality, at least 12 questions must be answered with a ‘Yes.’

**Chart 1:** NHLBI Quality Assessment Tool for Observational Cohort and Cross-Sectional Studies^(22)^

1. Was the research question or objective in this article clearly stated?
2. Was the study population clearly specified and defined?
3. Was the participation rate of eligible individuals at least 50%?
4. Were all subjects selected or recruited from the same or similar populations (including the same time period)? Were inclusion and exclusion criteria for being in the study prespecified and applied uniformly to all participants?
5. Was a justification of sample size, description of power, or estimates of variance and effect provided?
6. For the analyses in this article, were the exposure(s) of interest measured before the outcome(s) were measured?
7. Was the time period sufficient for one to reasonably expect to see an association between exposure and outcome, if one existed?
8. For exposures that can vary in amount or level, did the study examine different levels of exposure in relation to the outcome (e.g., exposure categories or exposure measured as a continuous variable)?
9. Were the exposure measures (independent variables) clearly defined, valid, reliable, and implemented consistently across all study participants?
10. Were exposure(s) assessed more than once over time?
11. Were the outcome measures (dependent variables) clearly defined, valid, reliable, and implemented consistently across all study participants?
12. Were the outcome assessors blinded to the exposure status of participants?
13. Was loss to follow-up after baseline 20% or less?
14. Were key potential confounding variables measured and adjusted statistically for their impact on the relationship between exposure(s) and outcome(s)?

Regarding the results analysis, information on authorship, publication year, journal, place/country of study origin, evaluated age group, sample size, objectives, findings, assessment tools, and main outcomes were extracted from the studies and organized in a Microsoft Excel spreadsheet. This organization facilitated a deeper discussion on the topic, enabling the analysis of behavioral and electrophysiological assessments of CAP in children and adolescents undergoing various musical training. It is important to mention that the data from the studies was analyzed descriptively and comparatively, as it did not support a meta-analysis. Hence, one can observe that an inferential analysis of the meta-analysis type would be interesting and that the form of analysis presented here limits the statistical robustness of the conclusions, although it does not invalidate the proposed reflections.

## RESULTS

**Figure 1:**
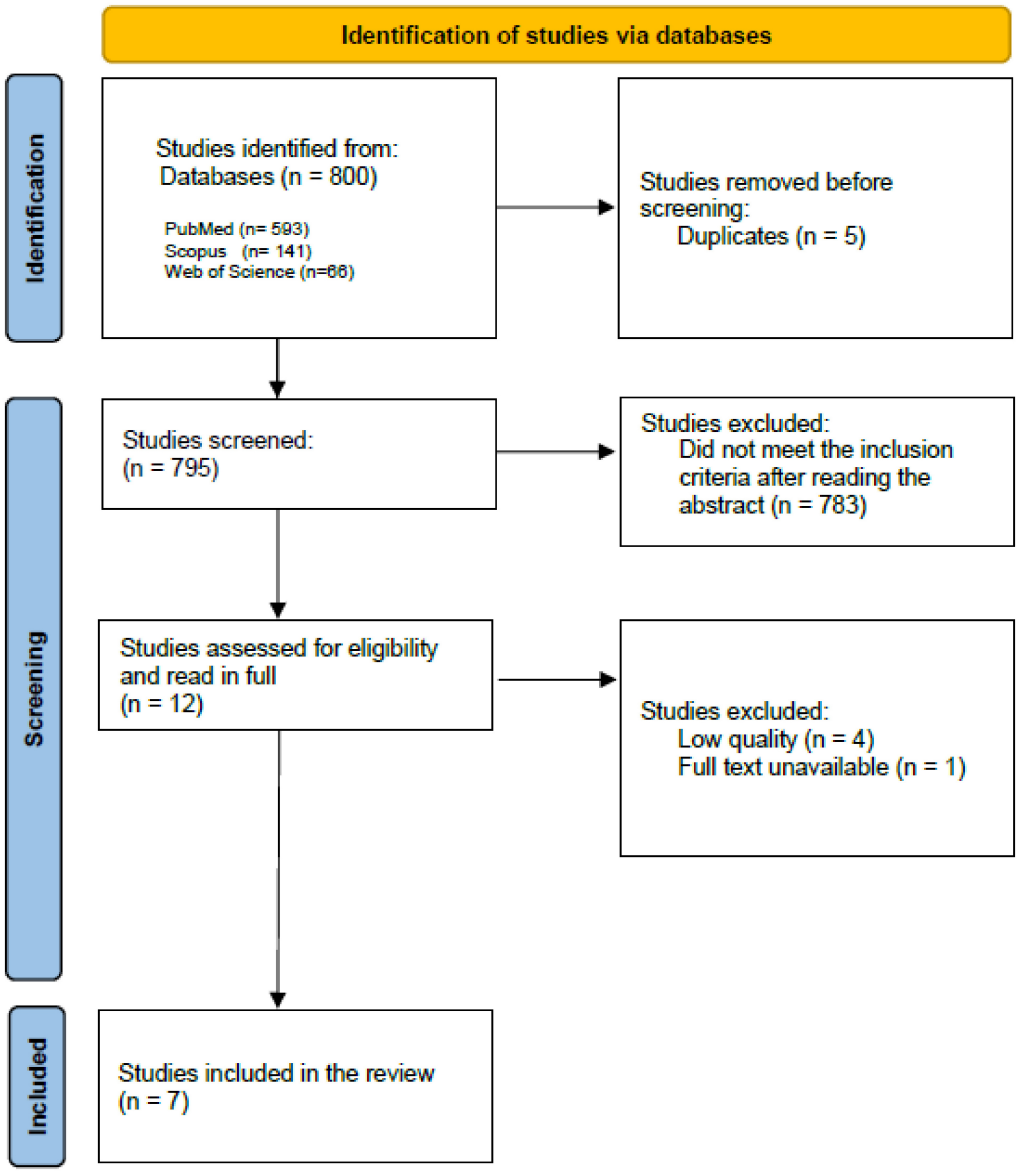
Flowchart of the search process, selection criteria, and analysis of the articles. Source: Adapted from PRISMA

Initially, the search yielded 800 publications, with 593 articles found in the electronic database PubMed, 141 in Scopus, and 66 in the Web of Science, with five being rejected due to duplication. This left 795 articles for title and abstract examination, of which 783 did not meet the inclusion criteria, that is, they were not in accordance with the target population, with the type of intervention in question, and/or with the selected study designs for this review. The analysis proceeded with 12 studies that were fully read. Upon further inspection, one article was excluded because the full text was unavailable; an additional four were excluded for poor quality, as they were deemed fair or poor based on the NHLBI Quality Assessment Tool^(22)^. Consequently, seven articles were selected for this study. Figure 1 illustrates the article search, analysis, and selection process.

### Characteristics of the selected studies

All included studies were deemed to be of high quality and featured well-defined objectives, criteria for population selection, and assessment and intervention instruments^(22)^. To analyze behavioral and electrophysiological auditory findings in children and adolescents undergoing musical training, a summary of the data —including authorship, publication year, country of origin, sample size and age, objective, assessment instruments, and main results — was compiled (Chart 2).

**Chart 2:** A summary of the included authors, year of publication, country, research objective, sample size and age, and main results

Authors and country	Objective	Sample size and age	Intervention/exposure procedure	Testing procedure	Main results
Chen et al.^(23)^, Taiwan	Investigating whether previous musical training improves pitch perception in implanted children.	27 pre-lingual deaf children (5–14 years).	Training in listening, singing, reading sheet music, and playing a musical instrument.	Auditory discrimination test with sequential piano tones.	Auditory discrimination increases with musical experience, and the longer the musical training, the better the results are for this auditory skill.
Strait et al.^(24)^, USA	To define associations between musical training during early childhood and subcortical speech coding in silence and noise, speech perception in noise, and memory and attention.	31 children with normal hearing (7–13 years).	Musical experience in early childhood (children who have practiced consistently for at least four years musical activities, with at least 20 minutes at least five days a week)	Words in noise test; the hearing in noise test; frequency-following response with and without noise; auditory and visual memory tests	Musically trained children have better speech perception in competitive noise, as well as in auditory working memory and auditory and visual attention tasks. Neural coding of speech, in both silence and noise, is also better in this group.
Putkinen et al.^(25)^, Finland	To longitudinally investigate the development of auditory discrimination skills in musically trained and untrained individuals.	133 children with normal hearing (7–13 years).	Playing a musical instrument, practicing choir and orchestra, and studying music theory from the age of seven as part of the daily school curriculum.	Mismatch negativity and P3a.	The study provides strong evidence that musical training increases neural auditory discrimination and attention.
Slater et al.^(26)^, USA	Longitudinal evaluation of the effect of group auditory training on speech perception in noise.	38 children with normal hearing (average of 8 years).	Musical training with activities to develop fundamental skills in tuning and rhythm, vocal performance, and musical instrument practice, among others, was carried out twice a week, for 1 hour, for over 2 years.	The hearing in noise test.	Long-term musical auditory training provided clinically significant results in understanding speech in noise.
Petersen et al.^(27)^, Denmark	Evaluating the benefits of a short, intense musical training program for adolescent cochlear implant users.	11 pre-lingual deaf adolescents (15.6–18.8 years).	The music training program combines computerized exercises with rhythm training, singing, and ear training activities (20 hours, scheduled over six days and spread over two weeks).	Multi-feature music discrimination test, Dantale II test (lists of sentences in noise adapted for the Danish language), and mismatch negativity.	No neuronal and behavioral discrimination improvement was observed after the proposed musical training.
Habibi et al.^(15)^, USA	To investigate the effects of a musical training program on the auditory development of children compared to children with sports training and no training in a longitudinal way.	37 children with normal hearing (6–7 years).	Musical training (six to seven hours a week for two years).	Long latency auditory evoked potentials.	The musically trained children showed a greater ability to detect changes in the tonal environment and accelerated maturation of auditory processing compared to those in the other two non-musically trained groups.
Putkinen et al.^(17)^, Finland	To examine the maturation of selective attention in musically trained and untrained children and adolescents.	146 children and adolescents with normal hearing (10–17 years).	Individual instrument lessons, group music lessons, and music theory from age 7.	Mismatch negativity and P3a	The results provide new convergent behavioral and electrophysiological evidence of an intermodal paradigm for the accelerated maturation of selective attention in musically trained children and adolescents.

The selected articles were published across seven journals, each with a notable impact factor, representing research from five distinct work groups. The studies originated from developed countries, including Taiwan, the United States of America, Finland, and Denmark.

The study samples ranged from 11 to 146 children and/or adolescents, encompassing both those with normal hearing and those with prelingual deafness rehabilitated via cochlear implants^(15,17,23-27)^. The studies universally employed convenience samples due to the nature of the research question.

Regarding musical intervention types, one study featured structured musical auditory training totaling 20 hours, distributed over six days and two weeks, focusing on rhythm training, singing, and auditory training^(27).^The rest were based on school curricula, including instrument lessons, choir and orchestra participation, music theory, and music training projects aimed at low-income children, focusing on tone, rhythm, and timbre in combination with music production^(15,17,23-26)^.

The behavioral and electrophysiological examinations of central auditory abilities utilized various assessment instruments, including the auditory discrimination test with sequential piano tones at different frequencies (256 and 495 Hz) for pitch perception assessment (sample size: 27 subjects; number of studies that used this instrument: one), words in noise test (31 subjects; one study), hearing in noise test (69 subjects; two studies), musical multi-feature discrimination test (11 subjects; one study), Dantale II test (adapted for Danish) (11 subjects; one study), and electrophysiological assessments such as frequency-following response (31 subjects; one study), P300 (316 subjects; three studies), mismatch negativity (MMN) (290 subjects; three studies) and the P1, N1, P2, and N2 components of the long latency auditory evoked potential (LLAEP) (37 subjects; one study).

The outcomes of the selected studies largely demonstrated positive results following musical auditory training. These outcomes included improved sound discrimination in both children using cochlear implants with prelingual deafness and those with normal hearing^(23,25)^, enhanced speech understanding of noise^(24,26)^, and favorable changes in the functionality of the central auditory pathway in children and adolescents^(15,25)^.

## DISCUSSION

The comprehensive review of studies demonstrates that musical training significantly benefits the auditory capabilities of children and adolescents. Extensive musical engagement not only improves auditory discrimination and speech perception in noisy environments but also accelerates the maturation of various central auditory processes. These findings highlight the potential of music as a therapeutic tool for auditory rehabilitation.

The initial selection process for this review identified a significant number of studies, with 800 articles sourced, reflecting the scientific community's growing interest in the therapeutic applications of music within the auditory/language domain. Although the final selection was significantly narrowed based on strict inclusion criteria, the quality of the resulting studies was commendable, as presented in Figure 1 and Table 1. The analyzed articles encompassed children and adolescents (aged 5–18 years), a critical period for the development and maturation of central auditory faculties.

**Table 1:** Quality analysis of the selected studies (n = 7) using the Study Quality Assessment Tool from the National Heart, Lung, and Blood Institute (2021)^(22)^

Year	Authors	Q1	Q2	Q3	Q4	Q5	Q6	Q7	Q8	Q9	Q10	Q11	Q12	Q13	Q14	Quality
2010	Chen et al.^(23)^	Y	Y	Y	Y	Y	Y	Y	NR	Y	Y	Y	NR	Y	Y	Good
2012	Strait et al.^(24)^	Y	Y	Y	Y	Y	Y	Y	NR	Y	Y	Y	NR	Y	Y	Good
2014	Putkinen et al.^(25)^	Y	Y	Y	Y	Y	Y	Y	NR	Y	Y	Y	NR	Y	Y	Good
2015	Slater et al.^(26)^	Y	Y	Y	Y	Y	Y	Y	NR	Y	Y	Y	NR	Y	Y	Good
2015	Petersen et al.^(27)^	Y	Y	Y	Y	Y	Y	Y	NR	Y	Y	Y	NR	Y	Y	Good
2016	Habibi et al.^(15)^	Y	Y	Y	Y	Y	Y	Y	NR	Y	Y	Y	NR	Y	Y	Good
2021	Putkinen et al.^(17)^	Y	Y	Y	Y	Y	Y	Y	NR	Y	Y	Y	NR	Y	Y	Good

**Caption:** Q = question; Y = yes; NR = not reported

It is crucial to emphasize that childhood and adolescence are critical windows for neuroplasticity, during which auditory experiences can profoundly influence the structural and functional development of the CANS^(28,29)^. The findings from this review are consistent with the broader literature, which suggests that targeted auditory experiences, such as musical training, can activate and refine neural connections, thereby enhancing CAP.

The fundamental principles of auditory training programs, including attention, memory, positive reinforcement, and progressive challenge^(30)^, are equally applicable to musical training. However, the variability in training protocols across studies makes direct comparisons challenging. Petersen and collaborators^(27)^ provided detailed information on a structured musical auditory training program, detailing the number of sessions, their duration, and specific activities. The lack of standardized training protocols in other studies limits the generalizability of the findings and underscores the necessity for future research to adopt more consistent methodologies. In the literature discussing musical activities specifically developed for CAP rehabilitation protocols – that is, controlled auditory training protocols – such activities are primarily available for adults who use hearing aids^(31,32).^

The articles included in this review indicate that musical training, as part of academic activities, has been positively received by some researchers. They contend that such studies effectively bridge the gap between science and everyday life by demonstrating the impact of musical training on participants' communication skills^(26)^. Notably, two studies were conducted in Finland^(17,25)^, a country renowned for its exemplary educational system^(33)^. This accentuates the stark contrast between the significant emphasis on music education in Finnish schools and the notably limited music education in Brazilian public schools^(34)^.

In Brazil, despite legislative efforts, music education remains scarce. Law no. 11.769/08^(35)^ mandates the inclusion of music as curricular content in schools, yet its implementation has been inconsistent. The positive outcomes of this review underscore the necessity of integrating musical training into school curricula to bolster auditory and language skills^(5,18,28)^. Hence, expanding access to music education could reconcile scientific evidence with educational practice, offering potential benefits to a wider demographic of Brazilian students.

Regarding intervention groups, five studies divided participants into two to three groups^(15,17,24-26)^, consistently featuring one subjected to musical training and another not, or engaged in alternative activities such as sports^(25)^. One study compared two groups undergoing musical training: one comprised of children with cochlear implants, the other of normal-hearing children^(27)^. Only one study focused on a single group of children, conducting solely intra-group analyses^(23)^. Various behavioral methods have assessed the impact of musical training on children and adolescents, many of which deviate from standard Brazilian clinical practice. Nevertheless, these tests have effectively shown that musical training can significantly benefit the rehabilitation of CAP within this demographic.

As for the impact of musical training on specific auditory skills, two studies explored its effects on speech perception in noisy environments using the Hearing in Noise Test^(24,26).^ Both studies found that individuals with musical training displayed superior speech recognition in such environments. These findings suggest that musical training may enhance not just CAP skills but also real-world communication abilities^(36)^.

Conversely, one study using a musical multi-character discrimination test and the Dantale II test (a sentence-in-noise test adapted for Danish speakers) reported no significant behavioral improvements in discrimination among adolescent cochlear implant users after training^(27)^. The authors suggested that the limited training duration of just two weeks contributed to these outcomes.

The rest of the studies did not conduct specific behavioral auditory evaluations to assess the impact of interventions on auditory skills in children and adolescents; instead, they emphasized electrophysiological data^(15,25)^.

The impact of musical training on the recruitment of new neural resources was evident, as demonstrated by various electrophysiological hearing tests. Five selected studies have reported electrophysiological findings in children and adolescents with musical training, showing a positive effect of musical training on P300 and MMN amplitude, P1/N1 peaks, and auditory responses evoked by the syllable /da/^(15,24,25,27)^.

Only one study found no change in neural responses following musical stimulation, attributing this absence of change to the short duration of the training program^(27)^. A longitudinal study examining the development of auditory discrimination skills in musically trained versus untrained children from school age to early adolescence, using LLAEP-P300 and MMN, provided robust evidence for the role of training in enhancing neural auditory discrimination and attention. This was evidenced by an increase in P300 and MMN amplitudes in the trained children over time^(25)^.

The increase in LLAEP amplitude correlates with the extent of synaptic activity during the perceptual processing of acoustic stimuli^(37-39)^. Moreover, analysis of LLAEP amplitude can enhance our understanding of cortical auditory processing and cognitive skill responses, elucidating physiological processes underlying attention, discrimination, and auditory memory^(37)^. Children and adolescents who have received musical training also excel in audiovisual selective attention tasks, exhibiting superior responses in the N1/MMN and late P300 to novel sounds^(15)^.

In a related study comparing neural coding of speech, presenting the syllable /da/, which lasted 170ms, in a noisy environment to children with and without musical training, findings showed that although both groups had similar responses in a silent setting, musically trained participants displayed superior performance when the /da/ stimulus was presented alongside background noise. This comparison of response components in silence versus noise revealed that the musically trained group had faster response times in noisy conditions than their non-trained counterparts. Notably, analyzing neural coding using the syllable /da/ is crucial for examining the Frequency-Following Response^(40)^. These responses are significantly influenced by activity in the auditory cortex, cochlear nucleus, inferior colliculus, and medial geniculate body^(41)^.

In a 2016 study comparing children undergoing musical training to their peers participating in sports training or not engaging in any systematic training, the music group exhibited a decrease in P1 amplitude, an increase in identifiable N1 component incidence, and an enhanced N1/P1 ratio from the start to the second year of training. Additionally, this group demonstrated reduced P1 peak latency compared to the sports group^(15)^. In the discrimination task, children engaged in musical training showed significantly improved precision in detecting pitch deviations, accompanied by a pronounced increase in the P3 component amplitude in response to these changes^(25)^.

An important aspect of this discussion concerns the reviewed studies that involved children and adolescents with cochlear implants^(23,27)^. Cochlear implants are electronic devices designed to simulate the function of damaged or absent hair cells by directly stimulating auditory nerve fibers. This allows individuals with severe or profound hearing loss to perceive sounds, especially speech^(42,43)^. Among this group, results varied: one study reported enhanced auditory discrimination following musical experience^(24)^, yet another study, employing short-term musical training, failed to yield any significant results^(27)^. This discrepancy likely stems from the duration of musical involvement. The latter study only involved a brief two-week period of musical training which incorporated computerized exercises, rhythm training, singing, and auditory tasks. Despite the inclusion of gamification to enhance attention, motivation, and therapeutic engagement^(44)^, 20 hours of musical stimulation were insufficient to replicate the advances reported in other studies, even for children with similar hearing profiles. This underscores previously mentioned findings on the importance of both the frequency and duration of training for effective rehabilitation.

Generally speaking, regardless of whether musical training is provided within a structured auditory protocol or in academic settings, a majority of the studies^(15,17,23-26)^ suggest that such interventions have a positive influence on various auditory-related facets. These outcomes, covering both behavioral and electrophysiological measures of CAP, are critical for daily and academic activities, supporting the development of lifelong skills in children.

One limiting factor of the study was the inability to perform an inferential analysis (e.g., meta-analysis) owing to the small sample size of studies and their heterogeneous results. Therefore, future research should aim to explore this topic using such analytical methods to derive more definitive conclusions. The discussions presented herein support the argument that Brazilian education would greatly benefit from the implementation of legislation mandating comprehensive music education in basic education.

## CONCLUSION

This review demonstrated that children and adolescents undergoing musical training exhibit improved behavioral and electrophysiological outcomes in central auditory processing. These improvements encompass enhanced sound discrimination, speech processing amid noise, detection of environmental sounds, and selective attention. However, it is critical to acknowledge that attaining significant results requires meticulous attention to the duration of the training.
